# Reliability of the Smartphone Application Inclinometer and Flexicurve in Measuring Thoracic Kyphosis

**DOI:** 10.7759/cureus.35886

**Published:** 2023-03-08

**Authors:** Gönül Elpeze, Günseli Usgu, Sedat Yiğit

**Affiliations:** 1 Department of Physical Therapy and Rehabilitation, Kalyon Medical Center, Gaziantep, TUR; 2 Department of Physical Therapy and Rehabilitation, Faculty of Health Sciences, Hasan Kalyoncu University, Gaziantep, TUR; 3 Department of Physical Therapy and Rehabilitation, Faculty of Health Sciences, Gaziantep University, Gaziantep, TUR

**Keywords:** thoracic kyphosis, inter-rater reliability, intra-rater reliability, smartphone, flexicurve

## Abstract

Introduction: This study examined the inter- and intra-rater reliability of the smartphone inclinometer app (SPI) and flexicurve (FC) for assessing the kyphotic angle in individuals with thoracic kyphosis (TK).

Methods: This study was conducted with 60 subjects (35 males, 25 females) aged 18 to 25 who presented to Kalyon Medical Center, Gaziantep, Turkey, between December 2021 and March 2022. The subjects were evaluated by two independent assessors using FC and SPI to measure the TK angle. The intraclass correlation coefficient (ICC) was analysed at a 95% confidence interval. The level of agreement between the methods was checked using Bland-Altman analysis.

Results: Inter- and intra-rater measurements were strongly correlated (ICC 0.945 and 0.964, respectively). On the Bland-Altman plots, the FC showed poor agreement with the SPI app (mean difference, 19.81° ± 2.8°). The mean kyphotic angles were 45.15 ± 6.07° and 25.34 ± 4.96°, respectively, as measured by the SPI and FC.

Conclusion: This study demonstrated good intra- and inter-rater reliability of the SPI app and FC for the measurement of the spinal curvature (TK) angle in the sagittal plane. A weak agreement was discerned between the SPI and FC methods.

## Introduction

Thoracic kyphosis (TK) is defined as the forward curvature of the spine between T1 and T12 in the sagittal plane. Angular values between 20° and 40° are considered normal; however, the incidence of TK increases with age. An increase in this curvature is termed hyperkyphosis, postural kyphosis or increased kyphosis [1]. Hyperkyphosis can occur at any age because of greater physiological curvature due to postural habits or age [2].

The clinical assessment of TK is very important for evaluating the functional and physical consequences of TK and the risk factors for its progression. Several methods are available for the measurement of TK, which can be classified as radiographic (RG) and non-radiographic (non-RG) [3]. The Cobb angle, an RG method, is accepted as the gold standard for the measurement of TK. Manual and digital inclinometers, flexicurve (FC) and smartphone inclinometer (SPI) are non-RG methods used for TK measurement in the clinical setting. The effective and widespread use of RG methods in the clinical setting is disputed due to many factors, including their high cost, limited portability, time-consuming nature and exposure to ionising radiation [4]. Consequently, researchers are developing inexpensive, easy-to-use and accessible non-RG tools and methods for assessing spinal curvatures in the clinical setting. Reliability and validity studies are available in the literature for non-RG methods, such as FC and Cobb angles and manual and digital inclinometers in TK measurement [5]. In previous studies, high levels of inter- and intra-rater reliability were reported for FC and other methods [6,7]. Excellent correlation with the Cobb method was demonstrated in a study examining the validity and reliability of the SPI method in TK measurement [8].

The procedure for applying the FC material to measure and calculate the TK angle is complex and time-consuming for the assessor. However, in the clinical setting, FC offers the advantages of accessibility and affordability compared to other methods. It is important for clinicians to be able to perform TK angle measurements and monitor the progression of the TK angle in a simple way in the clinical setting. Several types of inclinometers used to measure kyphosis produce fast results due to their easier application and simpler calculation of the TK angle. The SPI combines quick and simple angle calculation features of the inclinometers with the accessibility and affordability of FC for TK measurement. Thus, the current study sought to examine the reliability of the SPI app in TK measurement and compare the SPI app with the FC method’s demonstrated reliability and validity. This is the first study to report on the inter- and intra-rater reliability of SPI and FC methods for measuring TK curvature in the sagittal plane.

## Materials and methods

Participants

After obtaining approval from the Ethics Committee for Non-Interventional Studies of Hasan Kalyoncu University’s Faculty of Health Sciences (27.12.2021; No: 2021/048), patients who gave written informed consent were included in the study.

This cross-sectional study was conducted with 60 subjects (35 males, 25 females) from 18 to 25 years of age who presented to Kalyon Medical Center, Gaziantep, Turkey between December 2021 and March 2022. The subjects were selected from among the students of the Department of Physical Medicine and Rehabilitation at Hasan Kalyoncu University. Subjects with a TK angle ≥30º were included in the study. The TK angle required for inclusion in the study was measured using a bubble inclinometer (Baseline, Fabrication Enterprises Inc., NY, USA). Individuals were excluded if they had rigid TK, scoliosis (Cobb angle >10°), congenital spine abnormalities, shoulder, pelvic and other spinal injuries and medical treatments that impaired physical activity. In addition, professional athletes were excluded. Three subjects who did not return for the second measurement were also excluded. Ultimately, the study was completed with 60 subjects in total. The sample size was determined by considering similar articles in the literature [8-10]. Salamh et al. calculated the sample size for their study and reported that 28 subjects would be sufficient to achieve 80% power at an alpha level of 0.05 [10]. Therefore, 60 asymptomatic subjects met the inclusion criteria and participated in the current study.

Procedure and assessments

The physical characteristics (height, body weight and BMI) of the subjects were obtained. Measurements were taken by two experienced physiotherapists with at least five years of working experience in spinal health (raters) using FC and the SPI app (Samsung, Clinometer Version 2.4, com.plaincode.clinometer, Android 2.3.2+, 2016-05-30). Two measurements were obtained three days apart for each subject. For intra-rater reliability, each rater performed three measurements, and for inter-rater reliability, each rater conducted measurements separately. All assessments took place under the same physical conditions (temperature and light) and at the same time of the day.

For the TK measurement, the subject was positioned in a neutral standing position. Anatomical landmarks (T1-3-T12) were marked as described in the literature [6]. After the subject was asked to flex their neck, C7 was palpated and the T1 spinous process underneath was marked. Self-adhesive skin markers were used to indicate spinous processes. The landmarks were marked by the same physiotherapist to ensure standardisation, and the other physiotherapist obtained measurements from these locations. These measurements were performed sequentially, with the subject retaining the same posture.

Flexicurve measurement

**Figure 1:**
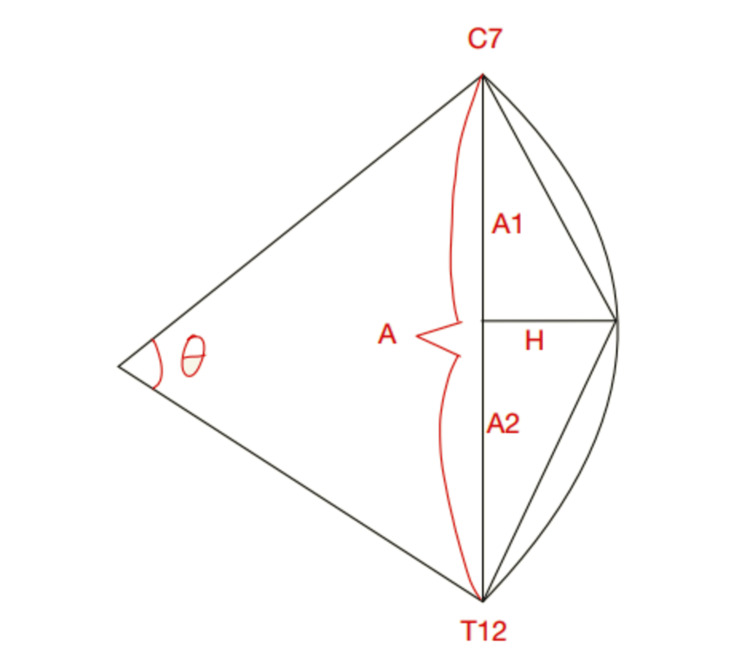
Flexicurve angle (Theta angle, 𝜃) is calculated using lines drawn perpendicular to the short sides of the triangle demarcated by the apex, cranial end of the curve and the caudal end of the curve. Kyphosis angle (𝜃) = (arctan (H/A1) + arctan (H/A2)).

The flexicurve is a ruler (40 to 80 cm long) which is a strip of flexible metal covered in plastic that retains its shape once bent. It measures the lumbar and thoracic curvatures in the sagittal plane. After waiting for about one minute for the subject to assume the normal posture, the FC was placed along these landmarks. Gentle pressure was applied to the FC to mould it to the spinal curvatures. The ruler was transferred onto white paper, taking care not to distort its shape and the curvatures were outlined with a pencil. The thoracic length (A) was measured in centimetres (cm) by drawing a straight line between the two ends of the ruler (C7 and T12). The thoracic height (H) in centimetres was determined by drawing a line from the apex of the thoracic curve to the straight line. The distances of the C7 and T12 points to the TH point were measured in cm as A1 and A2, respectively. The kyphosis angle was calculated using the following formula: arctan (H/A1) + arctan (H/A2) (Figure 1) [6,11].

Smartphone application

For the protractor software of the SPI to work, the short side of the phone was placed over the anatomical landmarks (T1-T3, T12) marked on the spine. First, the phone was placed on the T1-T3 spinous process and the protractor was set to 0°. Subsequently, the phone was placed on the T12 spinous process, and the angular value displayed on the screen was recorded as the kyphotic angle [8].

Statistical analysis

Data were analysed using IBM SPSS Statistics for Windows, Version 22.0 (IBM Corp., Armonk, NY, USA). Numeric data were summarised as mean ± standard deviation (X ± SD) and categorical data as a percentage (%). The normality of the data distribution was assessed using the Kolmogorov-Smirnov test. The significance level was set at 0.05. The correlation between curvature angles measured with FC and SPI was examined using Pearson’s correlation test, Student’s t-test (for pairwise comparisons) and intraclass correlation coefficient (ICC). Based on a 95% confidence interval, ICC values were interpreted as follows: very weak correlation if 0-0.20, weak if 0.20-0.39, moderate if 0.40-0.59, strong if 0.60-0.79 and very strong if 0.80-1.00 [12]. TK angles measured using each method were compared using the Bland-Altman analysis. Flexicurve TK angles scaled to the relevant angles were paired with SPI angles and evaluated for agreement on the data graph. The differences between the FC and SPI angle measurements (y-axis) were plotted against their means (x-axis) for each subject [13].

## Results

The subjects had a mean (± SD) age of 21.92 ± 1.50 years and a mean (± SD) BMI of 22.74 ± 3.46 kg/m^2^. Physical characteristics (age, body weight and BMI) are illuminated in Table 1.

**Table 1:** Physical characteristics of the study sample BMI: body mass index; X ± SD: mean ± standard deviation; min: minimum; max: maximum; m^2^: square meter; kg: kilogram

Physical Characteristics	Total (n=60)	
	X±SD	Min-Max
Age, years	21.92±1.50	17-24
Height, cm	174.42±9.95	154-194
Body weight, kg	69.58±14.23	24-95
BMI, kg/m²	22.74±3.46	7.41-31.64

Table 2 depicts the TK angles measured using SPI and FC. The mean kyphotic angles were 44.89°±6.21° and 25.23±5.56°, respectively, measured by SPI and FC for rater I (p>0.05). The mean kyphotic angles were 45.42°±6.33° and 25.44±5.10°, respectively, measured by SPI and FC for rater II (p<0.05). The mean TK angle from the FC measurements was about 20° lower than that measured by the SPI app.

**Table 2:** Mean thoracic kyphosis angles as measured by the smartphone app and flexicurve *p<0.05, FC: flexicurve, SPI: smartphone inclinometer, Wilcoxon Signed Ranks Test

	Rater I X±SD (min-max)	Rater II X±SD (min-max)	P
FC	25.23°±5.56° (13°-40°)	25.44°±5.10° (15°-42°)	0.379
SPI	44.89°±6.21° (31°-65°)	45.42°±6.33° (32.6°-66°)	0.010*

Very strong inter- and intra-rater correlations were found when analysing the measurement results obtained by two assessors using the SPI app. The inter-rater correlation between the SPI app and FC measurements was very strong (ICC = 0.964, ICC = 0.912). Similarly, a very strong intra-rater correlation level was found for both methods. The correlation between SPI and FC measurements was 0.961 and 0.969 for rater I, and 0.945 and 0.908 for rater II (Table 3).

**Table 3:** Results for inter- and intra-rater reliability SPI: smartphone inclinometer, FC: flexicurve, ICC: intraclass correlation coefficient

		p	Intra-rater	Inter-rater
			ICC (95% CI)	ICC (95% CI)
SPI	Rater I	<0.001	0.961 (0.933-0.976)	0.964 (0.94-0.97)
	Rater II	<0.001	0.945 (0.948-0.981)	
FC	Rater I	<0.001	0.969 (0.907-0.967)	0.912 (0.85-0.94)
	Rater II	<0.001	0.908 (0.846-0.945)	

**Figure 2:**
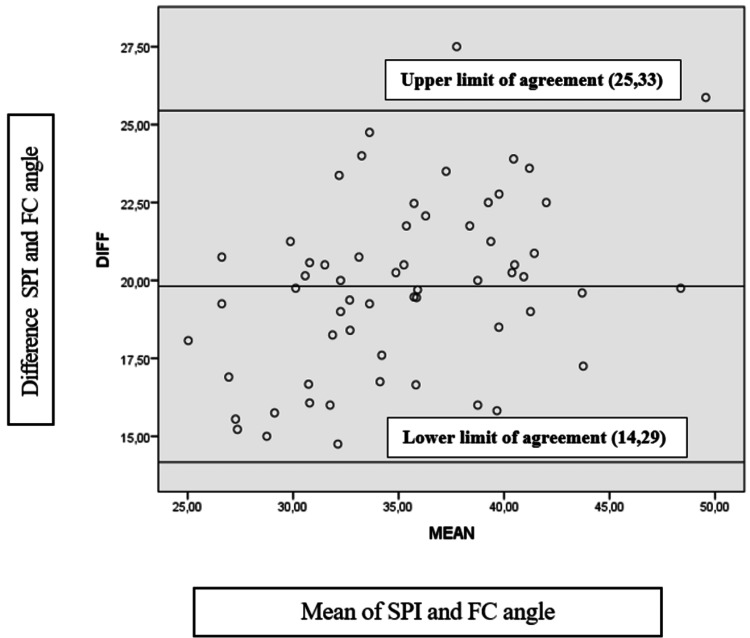
Bland-Altman plot of the smartphone app inclinometer angle and each of the flexicurve angles FC; flexicurve, SPI; smartphone inclinometer, DIFF; difference

The levels of agreement between SPI and FC angles are displayed in Figure 2 (near here) using Bland-Altman data plots. The mean of the differences between the SPI and the FC was 19.81±2.8° as shown in the graphic representation. The upper limit of agreement was 25,33° and the lower limit of agreement was 14,29. The plot demonstrated proportional bias (SPI and FC angle p=0,001). SPI demonstrated weak agreement with the FC.

## Discussion

This research examined the inter- and intra-rater reliability of SPI and FC methods to measure the TK angle. The SPI and FC methods revealed a very strong correlation. High levels of intra- and inter-rater reliability were found for both methods (ICC = 0.88). A weak agreement was discerned between the SPI and FC methods.

Spinal curvatures can be measured using RG methods that is the Cobb angle and (vertebral) centroid angle (CA) and non-RG methods that is FC, manual and digital inclinometers, motion capture, arcometer, flexible electrogoniometer and smartphone apps. None of these tools and methods is completely free of potential measurement errors [4]. Indeed, an error rate of ± 5º has been reported for the Cobb angle derived from a radiograph, considered the gold standard for the measurement of spinal curvatures [14]. Although reported Cobb angle errors are usually small, individual differences as large as 30° can be observed [15,16]. This error is due to the difficulty of accurately locating bony landmarks clearly on the radiographs [17]. Typically, palpation errors can also occur in non-invasive measurements of the spine. However, there is evidence from studies that the experience of the assessor can minimise palpation errors [18]. We believe that the margin of error in our study was reduced due to the years of experience of the two physiotherapists (21 and seven years) included as assessors in our study compared to other studies [6,19] and due to the palpation being performed by a single assessor.

Recently, clinicians have started using diverse inclinometer applications on smartphones for angular measurements of body parts. In the literature, only a few studies have reported the validity and reliability of the SPI app, a non-RG method. Most available studies have compared universal goniometry (used to measure joint ROM) with the SPI app [20,21]. Only one study compared the gold-standard Cobb method with the inclinometer app in TK measurement. Shahri et al. compared the Goniometer-Pro app with the Cobb angle method for the measurement of TK in 31 participants, as assessed by three raters. They found an excellent correlation (ICC = 0.81) between intra-rater (ICC = 0.88) and inter-rater reliability (ICC = 0.915). Both methods also demonstrated good agreement [8].

Moderate and strong correlations between FC and RG methods have been reported in many studies [4,6,22]. The validity and reliability of three non-RG methods (i.e. Flexicurve kyphosis angle, Flexicurve kyphosis index and Debrunner kyphosis angle) versus the Cobb method were examined in 113 individuals older than age 60 with a TK angle of <40°. A high correlation was reported between the Cobb angle and the FC (ICC = 0.67-0.76) [4,6]. A study involving 40 adults (22 females, 18 males) investigated the validity of RG and non-RG (FC and motion capture) methods in the measurement of kyphosis and illustrated a moderate correlation between the FC and Cobb methods (ICC = 0.403). In that study, which aimed to confirm the validity and reproducibility of FC use for the assessment of lumbar and thoracic curvatures, the authors found no statistically significant difference between FC and Cobb angle measurements. Inter- and intra-rater measurements were highly correlated (ICC = 0.72) [22]. Spencer et al. reported a high correlation (ICC = 0.61) between FC and the centroid (vertebral) angle (CA), which is an RG method [3].

Studies have revealed moderate and strong correlations between FC and non-RG methods [6,11]. In a study using FC and a manual inclinometer to measure the TK angle in 30 swimmers, investigators checked the level of agreement between the FC angle and manual inclinometer angle and sought to formulate an equation involving both angles. They reported good inter-rater reliability (ICC = 0.86) and excellent intra-rater reliability (ICC = 0.94) for FC [11]. High levels of inter- and intra-rater reliability were found for non-RG methods in the measurement of the TK angle (ICC = 0.96) [6]. Three evaluators reported excellent inter-rater reliability (ICC 0.93 and 0.94) for the FC method in measuring the TK angle in 51 subjects (21-88 years of age) [19]. Corroborating the aforementioned studies, we found very strong intra-rater (ICC = 0.90 to 0.96) and inter-rater (ICC = 0.91) correlations in our study. In the current study, the intra-rater results of one assessor were lower than the inter-rater results. This disparity can be explained by the two assessors obtaining measurements sequentially on the same day, without moving the participant. The second measurements were performed in the same way three days later.

In the studies, besides the reliability of the methods used for TK measurement, their agreement was investigated. The agreement indicates the degree to which scores are identical among objects or subjects [23]. Reliability is the ratio of variability between objects or subjects to the overall variability of all measurements in the sample. Therefore, reliability refers to the ability of a measurement to differentiate between objects or subjects [23,24]. As such, it can be said that the reliability and agreement results of a study provide information to the researcher about different aspects. Accordingly, a strong correlation between any two methods does not necessarily signify a strong agreement between them. The smaller the difference between the methods, the stronger the agreement [13]. In studies, the FC angular value has been systematically reported to be smaller than the angular values measured by Cobb and non-RG methods (mean difference, 20.3 ± 6.1°) [3,6]. This disparity between the methods has been explained by multitudinous factors. First, the FC tool may perform a fundamentally different angular measurement than the RG methods. Second, for the angular assessment of TK, it may not be appropriate to directly compare FC measurement results to the Cobb angle [6,9]. Third, the TK angle measured with the FC was calculated using a geometric formula. The use of scaling metrics specific to this geometric formula may be more applicable to a certain angle or population. In our study, the FC angular values were approximately 20.27° lower than the SPI angular values.

Correspondingly, smaller FC angular values compared to those obtained with other methods resulted in a weak agreement between the FC method and the other methods. Despite the strong correlation of FC with other methods, published studies report weak agreement between methods [25,26]. Barrett et al. compared FC and manual inclinometer methods among 11 subjects (seven males, four females). Referring to the Cobb angle, that study reported a strong correlation (ICC = 0.96) but a weak agreement between the FC angle and the Cobb angle [9]. Consistently, a weak agreement was found between SPI and FC in our study.

In one study, a method showing good agreement with FC in TK measurement was noted. Spencer et al. reported strong agreement between the FC method and the CA. The authors attributed this strong agreement to the similarities in the measurement techniques of both measurement methods [3]. In the CA method, the angular value is determined by measuring the intersection angle of two straight lines drawn perpendicular to the two uppermost and two lowermost vertebrae involved in the thoracic curvature. Like FC, the CA method measures the spinal contour using the T1-T12 vertebrae [27]. Like Cobb, CA is measured using spinal radiography in the sagittal plane [16].

Studies have not reported large angular differences between the Cobb angle and non-RG methods (except FC) in TK measurement. Thus, a strong agreement was noted between these methods [3,5,9]. We think that the very similar angular values observed with these methods relate to the similarity of the measurement technique and angular calculations, which are essentially based on geometry rules. The Cobb angle is measured in the sagittal plane using spinal radiography and recorded as the angle between the two lines drawn perpendicular to the tangential lines along the superior endplates of the last two vertebrae that contributed to the thoracic curvature [14]. In non-RG methods such as bubble, manual and digital inclinometers, the TK angle is recorded as the angular value indicated by the inclinometer placed over the T1-3 and T12 vertebrae [9,25]. Likewise, the SPI app used in the present study also measures the TK angle from the same vertebrae using the same technique. We believe the SPI measurement technique increases its agreement with other methods. Shahri et al.’s findings corroborate our results [8].

The mean TK is 44±11° in adults without spinal disorders, with a range of 19°-63° reported in 95% of the normal adult population [28]. The kyphosis angle has been reported to increase with age, and higher kyphosis angles have been associated with vertebral fractures, disc degeneration, and smaller, less dense trunk muscles [29]. The measurement of the TK angle and monitoring of its progression in young adults may be useful both in the management of spinal problems with onset in adolescence and in the identification of the risk factors for physical and functional consequences that may occur in old age. Considering the aforementioned data, we believe it is important to obtain robust data on the reliability of the FC and SPI methods used in our study to assess kyphosis in a healthy young population with a TK ≥30°. We believe that it would be useful to consider the agreement and correlation of the SPI method with the gold-standard method and other inclinometer methods, especially in clinical measurements. Given the advantages that the SPI method offers to the practitioner compared to FC, the SPI can be used to screen kyphosis in workplaces, primary healthcare facilities and schools. SPI offers a number of advantages to patients including no risk of side effects, very high reproducibility, no cost and readily accessible. It can be concluded that the SPI ideal and useful tool to use for the purposes of early diagnosis and following of TK for patients. Further studies are warranted to examine the validity and reliability of the SPI method compared to other non-RG methods and the Cobb angle in measuring TK. Also, the increase in mobile messaging and gaming and the use of smartphones have started to create important health problems. Researches revealed that the distinct cervical flexion seen in heavy smartphone users is causing a new overuse condition known as "text neck" [30]. Other segments of the spine can be investigated in future studies.

Limitations

Some limitations should be noted for the current investigation. First, repositioning errors were observed during three repeated measurements obtained during the same session. Second, the study measurements were performed three days apart, which may have caused postural awareness among the subjects. The TK angle measurement might have been affected by the pressure applied to the skin for the placement of the FC and SPI tools and to mould the FC to the curvature. Another limitation is that the Cobb method, the gold standard for TK measurement, was not used. Accordingly, the validity of the methods could not be evaluated. We believe that the lower intra-rater results compared to the inter-rater results noted for one of the assessors resulted from the two assessors’ performing the measurements consecutively on the same day without moving the participant. The sample size was not calculated using G*Power.

## Conclusions

This study showed that both the SPI app and the FC have good intra- and inter-rater reliability in the measurement of the TK angle in the sagittal plane. A weak agreement was discerned between the SPI and FC methods. While these non-radiographic measurements were applied in future clinical researches or practices, the low agreement between the two methods should be considered by clinicians.

The use of the SPI method for TK angle measurement may offer advantages including time-saving, cost-effectiveness and convenience. Consequently, SPI can be recommended as a method that clinicians can use for the evaluation and follow-up of TK.
